# Modulation of the dynamics and cellularity of adipose tissues in different fat depots in broilers by dietary dexamethasone

**DOI:** 10.5455/javar.2022.i627

**Published:** 2022-11-18

**Authors:** Nasrin Sultana, Rafiqul Islam

**Affiliations:** 1 Department of Anatomy and Histology, Faculty of Veterinary Science, Bangladesh Agricultural University, Mymensingh, Bangladesh

**Keywords:** Adipose tissue, adipocyte, broiler, dexamethasone, fat depot

## Abstract

**Objective:**

The objective of this investigation was to determine the effects of dexamethasone (DEX) on the weight and cellularity of abdominal and subcutaneous fat depots.

**Materials and Methods:**

The study was conducted on four broiler chicks (20 chicks per group) fed commercial feed and water ad libitum. The DEX was supplied with feed at 0 mg/kg (non-DEX), 3 mg/kg (DEX-1), 5 mg/kg (DEX-2), and 7 mg/kg (DEX-3) from day 0 to day 28. The entire abdominal and subcutaneous fat depots were collected and weighed after sacrificing five birds from each group on days 14 and 28.

**Results:**

The DEX groups had considerably lower (*p* < 0.05) fat depot weights with dose-related variation noted among the DEX groups. The histological findings revealed the presence of unilocular, round to oval-shaped adipocytes. The DEX-1 and DEX-2 had way lower (*p* < 0.05) numbers of adipocytes while the DEX-3 had considerably higher (*p* < 0.05) numbers of adipocytes than the non-DEX. DEX-1 and DEX-2 had larger (*p* < 0.05) adipocytes whereas DEX-3 had smaller adipocytes than the non-DEX. Adipocyte sizes and fat depot weights were found to have very strong negative relationships.

**Conclusion:**

Dietary DEX affects the growth and distribution of abdominal and subcutaneous fat depots and adipocyte cellularity subjected to both dose and duration of DEX treatment.

## Introduction

The poultry business has been expanding worldwide rapidly in recent years and is a significant contributor to agriculture-based economies. Every year, more than 66 billion broilers are slaughtered, according to the Food and Agricultural Organization database [1]. This agricultural subsector has grown sustainably for decades, driven by the constantly increasing demand for animal protein [2]. Broiler growth rates and meat productivity have improved in recent years due to their genomic modification and development programs. In addition, a high quantity of dietary energy is needed to get the broilers to market value, which has been reduced dramatically [3]. As a result of this unidirectional selection, multiple unwanted traits such as excessive fat deposition, higher risks of metabolic disorders, and increased mortality have emerged, causing consumer refusal of meat [4]. Moreover, these excessive fat depots have a profound impact on the nutritional quality of meat [5]. Fat deposition, particularly in the abdominal and subcutaneous regions, has become a major concern for the food processing industries since it also affects the further processing of broiler meat [6].

Currently, adipose tissue is considered an endocrine gland with a critical function in energy balance [7]. Adipose tissue is deposited in numerous body areas where the abdominal depot shows rapid post-hatch development [8]. Fat depot size in the abdomen is strongly related to the overall fat depot size since it accounts for about 2%–3% of the live weight and can be a practical selection trait for meat-purpose chickens such as broilers [4,9]. A rise in adipocyte number and size contributes to the cellular development and deposition of adipose tissue [10]. Age-related increases in adipocyte volume and number are positively correlated with the size of the fat depot and body mass [11]. In the initial phases, the increase in fat depot weight is mainly caused by an increase in the adipocyte population. But later on, the buildup of lipid particles in fat cells takes precedence, claims the study report previously cited [11]. Recent studies show that an increase in the size of adipocytes is mostly to blame for the increase in fat deposition in broilers at about 7 weeks of age [12].

The bulk of lipids stored in the adipose tissue is mostly of hepatic origin, circulated through the bloodstream, and deposited in the adipose tissue [7,13]. Adipose tissue is a major site for glucocorticoid (GC) receptors [14]. According to reports, GC therapy regularly promotes hepatic lipogenesis [15]. GCs naturally produced by the adrenal gland can increase fat storage in birds by promoting lipogenic activities in the hepatocytes [11]. The previous study report has shown that dexamethasone (DEX), a synthetic GC, increases hepatic lipogenesis, resulting in augmented fat deposition in broiler adipose tissues [16]. Dietary DEX supplementation also causes lipid droplet accumulation in broiler livers [17]. DEX induces preadipocyte differentiation and is engaged in adipocyte maturation [18]. However, DEX-induced fat deposition in different body regions is largely dose-dependent [19].

Research on adipose tissue has gained significant focus in the last few years for its role in weight gain in poultry and mammals [20,21]. Several studies have been conducted during this time to explain the impact of dietary nutrient density and composition, the sex-dependent distribution pattern of adipose tissue, and so on [22–24]. The broiler is an adequate animal model for studying adipose tissue due to its rapid growth rate and lipid deposition [24]. As far as we know, there is a lack of information depicting the effect of DEX on the histomorphometric traits of adipose tissues, particularly addressing the heterogeneity of subcutaneous versus abdominal fat depots in broilers. The development of adipose tissue is site-specific [25]. In this context, an elaborate study of the morphological attributes of different fat depots might provide a deep insight into the cellularity of these depots in broilers. Hence, we quantified the total amount of abdominal and subcutaneous fat depots’ average number of adipocytes and determined the mean adipocyte areas (μm^2^) of these fat depots to investigate the morphological changes in adipocyte number and size in response to DEX treatment.

## Materials and Methods

### Ethical statement

The institutional ethics committee of Bangladesh Agricultural University (BAU) granted permission for animal experimentation, which was carried out following the institutional ethical standards [Animal Welfare and Experimentation Ethics Committee-BAU-2020(3)].

### Research design

The experiment was conducted on healthy 1-day-old broiler chicks (N = 80) for 28 days. The chicks were randomly divided into four identical experimental groups, i.e., the control group (non-DEX) and DEX groups (DEX-1, DEX-2, and DEX-3), and allotted to individual pens. The broilers were maintained in identical conditions, with a balanced diet and drinking water (ad libitum). Notwithstanding this, dietary DEX (Decason, Opsonin Ltd., Bangladesh) was given to the DEX groups at rates of 3 mg/kg (DEX-1), 5 mg/kg (DEX-2), and 7 mg/kg (DEX-3) feed [19,26]. The diet’s composition is disclosed in a published publication [17].

### Sample collection

Five apparently healthy birds from each experimental group were sacrificed on the 14th and 28th days of the experiment to collect abdominal and subcutaneous adipose tissue depots. The collection of adipose tissue depots was done right away after the sacrificed broilers were dissected and weights (gm) were recorded.

### Histomorphology

After being fixed for 72 h with a 10% solution of buffer formalin (Merck, Germany), the fixed tissues were dehydrated with increasing grades of ethanol (Merck, Germany). The dehydrated tissues were then cleared with xylene (Merck, Germany), immersed in three grades of liquid paraffin (i.e., 58°C, 60°C, and 62°C), and finally embedded with paraffin wax (62°C). Five-micrometer (μm) thick sections were then prepared. For the histological study, the tissues were stained with routine hematoxylin-eosin stain (Merck, Germany). Finally, the slides were mounted with distyrene-dibutyl phthalate-xylene (Merck, Germany). The tissues were then blindly investigated, and the necessary images were captured. The number of adipocytes per microscopic field and their areas (the unit of measurement was square micrometer, μm^2^) from five randomly selected fields per tissue section was determined using the ImageJ freehand tool, a computer-assisted image analyzing software.

### Statistical analyses

An entirely random design was used in this experiment. Statistical Package for the Social Sciences (V-22) was used to analyze the data (IBM Corp., USA). The Shapiro–Wilk and Levene’s tests for variances were performed to analyze if the collected data for all variables had a normal distribution and homogeneous variance. The one-way analysis of variances with Duncan’s post hoc test was performed to find the differences among the study groups. A Pearson’s correlation coefficient test was done to measure the degree of relationship between the size of adipocytes and depot weights. A value *p* < 0.05 was considered significant, while *p* < 0.01 was considered highly significant.

## Results

### Weight of fat depots

The weight of two major fat depots of the broiler at different ages was compared in this study between the experimental groups. The results are shown in Tables 1 and 2. Compared to non-DEX, all DEX-supplemented groups had considerably (*p* < 0.01) lower amounts of abdominal fat. Noticeable differences (*p* < 0.01) between the DEX-supplemented groups were also evident. The DEX-1 had the maximum amount of abdominal fat while the DEX-3 had the lowest on days 14 and 28. The DEX-3 had a relatively lower (*p* > 0.05) abdominal fat depot weight than the DEX-2. In the case of subcutaneous fat depot weight, a non-significant (*p* > 0.05) numerical decrease was found between non-DEX and DEX-1. But the DEX-2 and DEX-3 had considerably (*p* < 0.01) lower subcutaneous fat depot weight than the non-DEX. The amount of both fat depots significantly increased (*p* < 0.05) on day 28 compared to day 14.

### Histomorphological study

**Figure 1.**
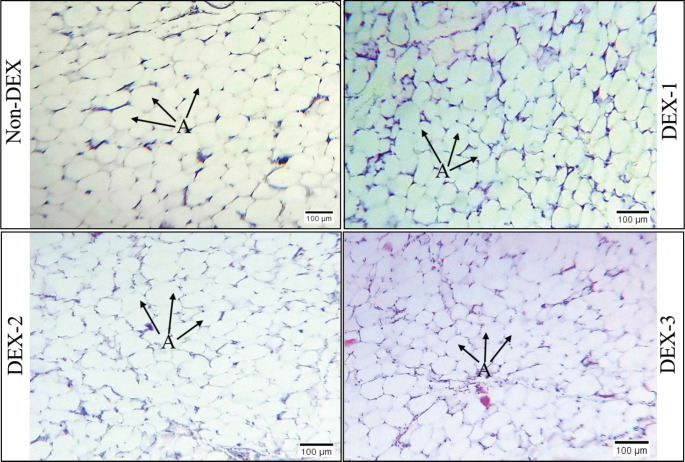
Histological images of the abdominal adipose tissue from non-DEX (DEX at 0 mg/kg feed), DEX-1 (DEX at 3 mg/kg feed), DEX-2 (DEX at 5 mg/kg feed), and DEX-3 (DEX at 7 mg/kg feed) groups of broilers. Hematoxylin-eosin staining. A – Adipocytes. Actual magnification 400×; scale bar = 100 μm.

**Figure 2.**
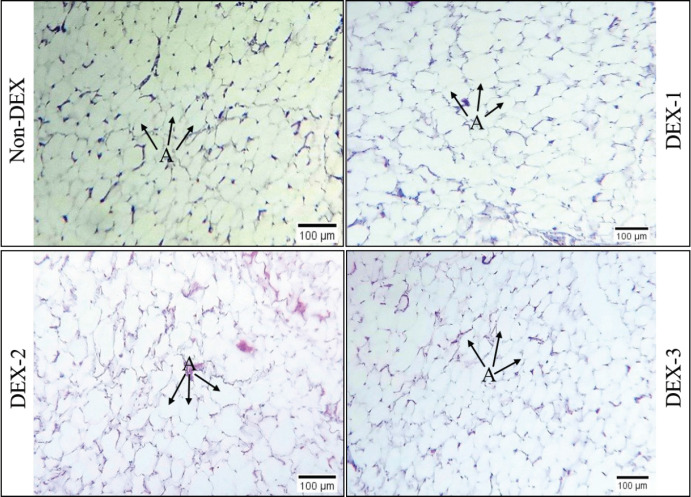
Histological images of the subcutaneous adipose tissue from non-DEX (DEX at 0 mg/kg feed), DEX-1 (DEX at 3 mg/kg feed), DEX-2 (DEX at 5 mg/kg feed), and DEX-3 (DEX at 7 mg/kg feed) groups of broilers. Hematoxylin-eosin staining. A – Adipocytes. Actual magnification 400×; scale bar = 100 μm.

In the course of our histomorphological examination, we noticed that the adipocytes in both fat depots were kept in place individually and were separated by thin fibrous septa that formed lobules inside the adipose tissue. In each fat depot, typical, unilocular, round to oval-shaped adipocytes were present. Within the fibrous septa, small blood vessels, including arteries and venules, were observed. The size of the adipocytes was somewhat larger in DEX-1 and DEX-2 than in non-DEX but relatively smaller and more concentrated in the DEX-3 group. The results are shown in Figures 1 and 2.

### Effects of DEX on the adipocyte population

The findings of the histomorphometric analysis are shown in Tables 1 and 2. DEX-1 and DEX-2 had considerably (*p* < 0.01) fewer adipocytes per microscopic field (400× magnification) in the abdominal fat depot. On the other hand, DEX-3 had a considerably (*p* < 0.01) higher abdominal adipocyte count per microscopic field. Similar findings were observed in the case of the subcutaneous fat depot, where DEX-1 and DEX-2 had considerably (*p* < 0.01) lower adipocyte counts per microscopic field, whereas DEX-3 had significantly (*p* < 0.01) higher adipocyte counts. These findings were consistent across both days 14 and 28.

### Effects of DEX on adipocyte size

The effects of DEX on adipocyte sizes are shown in Tables 1 and 2. On day 14, adipocyte sizes in the DEX-1 and DEX-2 were noticeably (*p* < 0.01) larger (1.4-fold and 1.2-fold, respectively) in the abdominal fat depot than in non-DEX. On the other hand, the DEX-3 had significantly (*p* < 0.01) smaller (1.2-fold) adipocytes compared to the non-DEX group on day 14. On day 28, adipocyte sizes in the various groups followed a similar trend, with sizes markedly (*p* < 0.01) increasing in the DEX-1 (1.5-fold), and DEX-2 (1.3-fold) while notably (*p* < 0.01) decreasing in the DEX-3 (1.2-fold) than the non-DEX. The size of the adipocytes was significantly (*p* < 0.01) larger on the 28th day compared to the 14th day.

**Table 1.** Weight (gm) of different fat depots, adipocyte count, and adipocyte size (μm^2^) in the non-DEX and DEX groups on day 14.

Item	Day 14			
	non-DEX	DEX-1	DEX-2	DEX-3
Depot weight (gm)				
Abdominal depot	6.18 ± 0.54^a^	4.20 ± 0.37^b^	2.55 ± 0.23^c^	1.48 ± 0.40^c^
Subcutaneous depot	2.91 ± 0.45^a^	2.73 ± 0.15^a^	1.53 ± 0.30^b^	0.80 ± 0.14^b^
Adipocyte count/microscopic field				
Abdominal depot	172.40 ± 5.50^b^	139.60 ± 4.21^c^	147.20 ± 3.56^c^	210.00 ± 4.8^a^
Subcutaneous depot	197.40 ± 3.97^b^	122.60 ± 3.01^d^	157.40 ± 3.61^c^	222.40 ± 5.74^a^
Adipocyte size (μm^2^)				
Abdominal depot	3545.13 ± 57.91^c^	4874.87 ± 62.65^a^	4339.60 ± 29.63^b^	3011.87 ± 42.65^d^
Subcutaneous depot	3386.90 ± 42.04^c^	5246.60 ± 36.85^a^	4234.40 ± 37.05^b^	2860.10 ± 42.54^d^
Depot weight vs adipocyte size (*p*-value)				
Abdominal depot	0.001			
Subcutaneous depot	0.075			

Values are presented as mean ± standard error of the mean (SEM).

^a,b,c,d^ Within a row, means with different alphabetic superscripts differ at *p* < 0.05.

**Table 2.** Weight (gm) of different fat depots, adipocyte count, and adipocyte size (μm^2^) in the non-DEX and DEX groups on day 28.

Item	Day 28			
	non-DEX	DEX-1	DEX-2	DEX-3
Depot weight (gm)				
Abdominal depot	24.33 ± 0.45^a^	6.44 ± 0.22^b^	3.63 ± 0.32^c^	2.93 ± 0.51^c^
Subcutaneous depot	7.14 ± 0.29^a^	5.04 ± 0.19^a^	2.62 ± 0.59^b^	2.37 ± 1.29^b^
Adipocyte count/microscopic field				
Abdominal depot	162.60 ± 3.61^b^	122.20 ± 2.85^c^	130.00 ± 2.49^c^	183.00 ± 4.37^a^
Subcutaneous depot	173.00 ± 4.18^b^	87.60 ± 3.47^d^	136.20 ± 3.29^c^	191.60 ± 8.55^a^
Adipocyte size (μm^2^)				
Abdominal depot	4032.07 ± 36.71^c^	5916.47 ± 33.18^a^	5112.47 ± 60.16^b^	3479.47 ± 36.54^d^
Subcutaneous depot	3873.70 ± 44.62^c^	6874.00 ± 45.47^a^	4924.70 ± 41.94^b^	3349.20 ± 27.93^d^
Depot weight vs adipocyte size (*p*-value)				
Abdominal depot	0.00003			
Subcutaneous depot	0.0002			

Values are presented as mean ± SEM.

^a,b,c,d^ Within a row, means with different alphabetic superscripts differ at *p* < 0.05.

On day 14, subcutaneous adipocyte sizes were substantially larger (*p* < 0.01) in the DEX-1 (1.5-fold) and DEX-2 (1.3-fold) than in the non-DEX. However, DEX-3 had significantly (*p* < 0.01) smaller (1.2-fold) adipocytes compared to the non-DEX group on day 14. Similar trends were observed on day 28, where subcutaneous adipocyte sizes significantly increased (*p* < 0.01) in the DEX-1 (1.8-fold) and DEX-2 (1.3-fold) groups but significantly decreased (*p* < 0.01) in the DEX-3 (1.2-fold) compared to the non-DEX. However, considerable (*p* < 0.01) variations were also found among the DEX-supplemented groups from days 14 to 28.

### Correlations between adipocyte sizes and different depot weights

As part of this study, we analyzed the correlations between the adipocyte areas and the weight of two major fat depots on days 14 and 28. The strength of association was measured between the mean adipocyte sizes and the weight of the entire fat depots. On day 14, the subcutaneous fat depot had no significant association (*r* = −0.407, *p* = 0.075, and *n* = 20) but the abdominal fat depot showed a strong reverse relationship (*r* = −0.662, *p* < 0.001, and *n* = 20) between the adipocyte size and depot weight. However, strong negative correlations were found in the cases of both abdominal (*r* = −0.794, *p* = 0.00003, and *n* = 20) and subcutaneous (*r* = −0.746, *p* = 0.0002, and *n* = 20) on day 28 (Tables 1 and 2).

## Discussion

Adipose tissue is the body’s major organ for storing energy and functions as an endocrine gland [7]. It is mainly distributed in the abdominal and subcutaneous regions of broilers [19,25]. An excess amount of fat is considered to be an economical loss as the bulk of it is discarded due to lower consumer acceptance. Hence, research on the growth and distribution of fat depots possesses greater significance in producing low-fat broilers since DEX increases hepatic lipogenesis and makes it easier for fat to build up in both the liver and adipose tissue, studying the differences in fat deposition and adipocyte morphology in different fat depots in broilers after long-term exposure to different doses of DEX may give us a clear picture of how this works.

Several factors affect how fat depots are distributed throughout the bodies of broilers [24]. In this study, we focused on how dietary DEX affected the development of two major fat depots in the broiler. Our results showed that the DEX-supplemented groups had significantly lower amounts of abdominal and subcutaneous fat. DEX plays a critical function in regulating energy metabolism by boosting catabolic activity and fuel utilization, which might explain the lower fat content in DEX-treated broilers [27]. Due to augmented energy waste [19,28], DEX significantly reduces feed efficiency in broilers. Relative fat content can be a crucial indicator for assessing DEX’s impact on adipogenesis, as DEX treatment suppresses the growth rate in the broilers [19]. Previous research found that DEX treatment significantly increases the relative fat depot weight (% of body weight) in broilers [16,19,28,29]. The increased deposition of body fat mostly happens in the liver of both human and avian species, and the insulin resistance resulting from the prolonged DEX therapy might be a potential reason for the increased deposition of body fat [16]. However, DEX’s effects are primarily determined by its dose and the type of fat depot [30,31]. The fat depot weight was much lower in the groups that received high DEX doses, indicating that DEX has a dose-dependent effect on fat accumulation. Such a dose-related outcome is possibly connected to lipolysis resulting from prolonged treatment with a high dose of DEX [32,33]. A similar observation was reported in the rat model, where a significant reduction in fat mass gain in response to DEX treatment [31]. Prolonged treatment with DEX increases the expression of the lipolytic genes in the fat depots, which justifies the current study findings [31]. In this study, we noticed the tendency of fat accumulation toward the abdominal region of the broiler, which agrees with the previous report [25]. A study on humans highlighted that DEX promotes fat accumulation in the abdominal visceral depots, indicating a similar pattern of DEX action in fat deposition in human and animal bodies [30]. Also, we found a strong link between the age of the broilers and the weight of the depots, which agrees with the earlier report [33].

The bulk of the adipose tissue is mainly made up of adipocytes and is affected by their size and quantity [25]. The number of adipocytes is regulated through the death and regeneration of new cells, whereas the size is regulated by lipid storage [23]. In this study, we found decreased numbers of adipocytes with increased adipocyte size in both abdominal and subcutaneous fat depots while DEX was supplied at a 3–5 mg/kg diet. Nevertheless, an increased number of adipocytes with decreased size was observed in the high-dose (7 mg/kg) group. This indicates a negative correlation between adipocyte number and adipocyte size that coincides with the earlier reports [24,34]. The variation in adipocyte sizes observed in different DEX doses indicates retarded development and maturation of adipocytes in the higher DEX dose group. In an earlier study, an increased number of adipocytes with decreased adipocyte size was reported in response to corticosterone implantation [33]. In another study, increased adipocyte size in the bone marrow was reported in the case of rabbit osteonecrosis induced by a steroid [35]. The GCs exert a more pronounced effect on the abdominal visceral depots than the subcutaneous ones [33]. However, adipocyte sizes of the subcutaneous depot were found to be comparatively more minor in all the groups compared to the abdominal adipocytes except the group supplemented with 3 mg/kg DEX. Another study [24] found a similar adipocyte size for both fat depots. In our study, we found significant negative correlations between adipocyte sizes and depot weights in the case of both fat depots. However, this mismatches the previous study findings where a strong positive relationship between adipocyte size and depot weight was reported [11,25,34]. An increased adipocyte population is considered healthier, whereas a decrease in adipocyte size results in death and inflammation [23]. However, in this study, we observed no inflammation in the adipose tissue.

GCs like DEX are frequently prescribed for treating different diseases in humans [36]. However, excess exposure to GC leads to augmented lipid accumulation in the visceral fat depots connected to insulin resistance, obesity, cardiovascular disorders, and different metabolic dysfunctions in either sex [31,37]. Therefore, the current findings might give a deep insight into the multi-dimensional relationship between the DEX dose, duration of DEX treatment, and fat depot weight as well as adipocyte cellularity. Understanding this relationship will help to develop strategies for using DEX in food animals as well as in humans. The results shown in this study will also provide a clear picture of the impact of GC supplementation on the production of healthy and safe broilers for consumers. In addition to the aforementioned discussion, it is noted that the current investigation was only focused on the main two fat depots in the broiler since adipose tissue is also a significant part of other tissues like muscle and liver. As a result, more research is needed to evaluate the role of dietary DEX on fat deposition in different organs, particularly in edible broiler tissues, and to study adipose tissue biology and its relationship to consumer health. 

## Conclusion

This study’s findings provide sufficient evidence that the impact of DEX on the growth and distribution of abdominal and subcutaneous fat depots and their morphologic and morphometric attributes in broilers is linked to both the dose and duration of exposure. Though this study found fewer fat depots in the abdominal and subcutaneous regions of DEX-treated broilers, the role of DEX in terms of growth rate and feed efficiency should also be considered.

## List of Abbreviations

BAU, Bangladesh Agricultural University; DEX, Dexamethasone; GC, Glucocorticoid; SEM, Standard error of the mean.
